# Lupus acute cardiomyopathy is highly responsive to intravenous immunoglobulin treatment: Case series and literature review

**DOI:** 10.1097/MD.0000000000025591

**Published:** 2021-05-07

**Authors:** Katya Meridor, Yehuda Shoenfeld, Oshrat Tayer-Shifman, Yair Levy

**Affiliations:** a Department of Internal Medicine E, Meir Medical Center, Kfar Saba; b Zabludowicz Center for Autoimmune Diseases, Sheba Medical Center, Tel HaShomer; c Rheumatology Unit, Meir Medical Center, Kfar Saba; d Affiliated with the Sackler Faculty of Medicine, Tel Aviv University, Tel Aviv, Israel.

**Keywords:** acute cardiomyopathy, intravenous immunoglobulins, myocarditis, systemic lupus erythematosus

## Abstract

**Introduction:**

Intravenous immunoglobulin (IVIg) is currently used with considerable success for the treatment of many autoimmune diseases, including systemic lupus erythematosus (SLE). Among its various indications, IVIg has also been found to be beneficial in myocarditis, whether or not it is associated with an autoimmune disease. Nevertheless, data regarding IVIg treatment for myocarditis/cardiomyopathy in patients with SLE are sparse. The objective of this case series was to describe our experience with IVIg as a treatment for lupus myocarditis and to review the literature for IVIg for this indication.

**Patient concerns:**

We report 5 female patients with SLE, who presented with signs of acute heart failure including pulmonary congestion and arrhythmias.

**Diagnosis:**

Echocardiography demonstrated new reduced left ventricular ejection fraction of 20% to 30%. Two patients underwent coronary artery angiography, which demonstrated normal coronary arteries, supporting the diagnosis of myocarditis or nonischemic cardiomyopathy.

**Interventions:**

High-dose IVIg treatment was initiated in all 5 patients.

**Outcomes:**

Following the treatment, clinical and echocardiographic improvement in cardiac function occurred within a few days to 1 month. This dramatic improvement persisted for several years.

**Conclusion:**

Based on our case series, we believe that IVIg has an important role in the management of lupus acute cardiomyopathy. This safe, well-tolerated optional treatment should be considered, especially in severe cases.

## 1 Introduction

Among the various treatment options for autoimmune diseases, IVIg is considered the mainstay of treatment for several conditions, especially Kawasaki disease and immune thrombocytopenic purpura. It is also used in the treatment of idiopathic inflammatory myopathies, antineutrophil cytoplasmic antibody vasculitis and autoimmune neurological conditions.^[1–3]^

In the last 2 decades, our group and others demonstrated the beneficial effect of IVIg treatment for SLE,^[4–11]^ with most data supporting amelioration of severe refractory flares and hematological manifestations following this therapy.^[9–15]^ Some report that IVIg is also effective in lupus nephritis,^[16,17]^ in neuropsychiatric manifestations,^[18–20]^ and during pregnancy.^[21]^

Cardiac involvement presents in up to 50% of SLE patients and pericarditis is the most frequent manifestation of SLE-related cardiac disease.^[22]^ However, all other cardiac components may be involved: endocardium, myocardium, conduction tissue, and coronary arteries.^[23]^ Lupus myocarditis (LM) is a rare but potentially fatal complication, affecting up to 10% of SLE patients.^[22,24–26]^ It may present as an acute illness or have a chronic course with the development of cardiomyopathy.^[26]^ The treatment of LM is generally empirical. Either oral or intravenous pulses of corticosteroids have been the mainstay of treatment, while cyclophosphamide, azathioprine, mycophenolate mofetil, and IVIg have also been used with some success.^[26,27]^ High-dose IVIg in SLE is mainly used as an adjunctive therapy when the standard treatments are ineffective or when immunosuppressive regimen is contraindicated. However, data concerning IVIg treatment for myocarditis/cardiomyopathy in lupus are sparse.

In this communication, we retrospectively review 5 cases who developed severe myocardial dysfunction, probably as a consequence of myocarditis secondary to SLE. All experienced dramatic improvement following IVIg therapy.

## 2 Cases

### 2.1 Patient 1

The details of this case of a 59-year-old female patient were described elsewhere.^[28]^ The patient presented to the Emergency Department (ED) with rectal bleeding. She had been diagnosed a few years earlier as having SLE, presenting with 4 of 11 American College of Rheumatology (ACR) criteria,^[29]^ including arthritis, pleuritis, high antinuclear antibodies (ANA) titers (1:1280), and elevated anti-dsDNA antibody titers. She was successfully treated with a few courses of IVIg and steroids for secondary myelofibrosis.^[15]^ Two months before admission, the patient had begun to receive 40 mg prednisone daily, which was continued throughout her admission. Upon admission, the patient was tachycardic, her blood pressure was 90/40 mm Hg, hemoglobin was 3.0 g/dL, white blood cell (WBC) count was 15.9 × 10^9^/L and platelet count was 587 × 10^9^/L. Both prothrombin time and partial thromboplastin time were within normal ranges and an electrocardiogram was unremarkable. Gastric suction demonstrated “coffee ground” appearance of the gastric contents. Angiography of the mesenteric vessels demonstrated a bleeding gastroduodenal artery. Consequently, embolization of the bleeding vessel, in addition to transfusion of 4 units of packed red blood cells were instrumental in stabilizing the patient's condition and achieving a hemoglobin of 9.6 g/dL.

Two days later, she developed a slow ventricular tachycardia and subsequently a ventricular fibrillation. After a successful resuscitation, she was transferred to the intensive care unit (ICU), where ST segment elevations were found in leads II, III, aVF, and V1–V6. Echocardiography demonstrated severe left ventricular dysfunction, with an akinesia that involved most of the left ventricle (LV) except for the basal segments. Estimated left ventricular ejection fraction (LVEF) was 20%. Creatine phosphokinase was 884 U/L (normal: 20–200 U/L) and its MB fraction was 147 U/L (normal: 5–25 U/L). A coronary angiography demonstrated normal coronary arteries. Hence, the differential diagnosis included acute myocardial infarction, either due to a thromboembolic event or vasculitis, vs myocarditis secondary to SLE. The patient refused to undergo cardiac biopsy. Therefore, the diagnosis of myocarditis was not proven histologically. The patient had no clinical signs of skeletal myositis. Of note, although she had arthralgia, anti-dsDNA, and antiphospholipid antibodies were negative during this hospitalization. Additionally, her ANA titer was 1:640, erythrocyte sedimentation rate was 90 mm/h and the antibody titers to SS-A, SS-B and RNP, and levels of C3 and C4, were within normal ranges. Three days later, the patient was treated with nitrates, histamine receptor blockers, angiotensin-converting enzyme inhibitors, thiamine and her regular dose of prednisone (40 mg/day). In addition, high dose IVIg treatment was instituted: she received 0.5 g/kg IVIg per day for 4 consecutive days. Neither anticoagulants nor antiaggregates were added, because of the threat of recurrent massive gastrointestinal bleeding. Two days after IVIg treatment was initiated, an echocardiography revealed no significant change; whereas, 1 week afterwards, the estimated LVEF increased to 30%. Three days later, the LVEF increased to 40%. The echocardiographic changes were associated with the patient's subjective feeling of improvement, as well as objective signs such as normalization of blood pressure and complete normalization of her electrocardiogram (without the appearance of Q waves). There was no change in the ANA titer, while the erythrocyte sedimentation rate decreased to 70 mm/h.

The patient was discharged 2 days later, and follow-up echocardiography 1 month later demonstrated normal LV function, with an estimated ejection fraction of 55%. No clinical or echocardiographic evidence of cardiomyopathy was found during the next 2 years.

### 2.2 Patient 2

A 34-year-old woman was hospitalized in the Department of Internal Medicine because of fever. SLE had been diagnosed 5 years previously, when she presented with 6 of the 11 ACR criteria^[29]^ (arthritis, malar rash, oral ulcers, direct Coombs’ positive autoimmune hemolytic anemia, high titers of ANA (1:1280) and elevated anti-dsDNA antibody titers). She had been treated regularly with 10 mg/day of prednisone and 200 mg hydroxychloroquine twice daily.

On admission, the patient was tachycardic, her blood pressure was 110/70 mm Hg, and her temperature was 38°C. Hemoglobin was 9.5 g/dL, WBC count was 11.3 × 10^9^/L, platelet count was 190 × 10^9^/L. Her electrocardiogram was unremarkable. On the fourth day of hospitalization, the patient developed progressive dyspnea. Bibasillar pulmonary crackles were present on auscultation. An emergency echocardiography demonstrated moderate to severe LV dysfunction, with widespread akinesia of the LV except for the basal segments, with an estimated ejection fraction of 25%. Based on our previous experience, we initiated high dose IVIg therapy. The patient received 1 g/kg IVIg for 2 consecutive days with rapid improvement in cardiac function. After 3 days, her LVEF improved remarkably to 55%. No evidence of cardiomyopathy was found during the next 3 years.

### 2.3 Patient 3

A 51-year-old woman with a common variable immunodeficiency syndrome associated with SLE and antiphospholipid syndrome was hospitalized in the Department of Internal Medicine due to dyspnea. She was diagnosed 4 years previously with SLE based on 4 of the 11 criteria^[29]^ (serositis, arthritis, positive ANA and positive anti-dsDNA antibodies with low C3 and C4 levels). She was treated regularly with IVIg (0.4 g/kg once a month). Upon admission, the patient was tachycardic, her blood pressure was 95/70 mm Hg. Hemoglobin was 11.0 g/dL, WBC count was 15.9 × 10^9^/L, and platelet count was 222 × 10^9^/L. An electrocardiogram demonstrated atrial fibrillation with left bundle brunch block. Echocardiography demonstrated moderate global LV dysfunction, with an estimated ejection fraction of 30%. Coronary angiography demonstrated normal coronary arteries. Based on our experience with IVIg therapy and due to her common variable immunodeficiency syndrome, we decided not to treat this patient with immunosuppressive therapy, but rather with high dose IVIg. The patient was given 0.4 g/kg IVIg per day for 5 consecutive days. After 2 weeks, her LVEF increased to 40%. One month after treatment, LVEF was 55%. The patient's clinical condition paralleled the echocardiographic improvement. No clinical or echocardiographic characteristics of cardiomyopathy were noted during the next 4 years of follow-up.

### 2.4 Patient 4

This 36-year-old woman with a past medical history of SLE and antiphospholipid syndrome was diagnosed 16 years earlier by presenting with 4 of the 11 ACR criteria^[29]^ including: arthritis, photosensitivity, high titers of anticardiolipin antibodies, anti-dsDNA antibodies and ANA (1:640). She had been treated with mycophenolate mofetil and warfarin due to recurrent thromboembolic events (3 events of deep vein thrombosis and an incident of pulmonary embolism). Additionally, she had a history of depression and 1 suicide attempt and was being treated with tricyclic antidepressants.

She was initially admitted with fever, chills, and a rash on her chest and neck, and was discharged once her symptoms subsided. Several days later she returned to the ED and was noted to have a superficial chest vein distention. She was diagnosed with a left jugular vein thrombosis and treatment was immediately begun with low molecular weight heparin. She was subsequently discharged home.

On her third admission, she complained of abdominal pain and fever. An abdominal CT demonstrated retroperitoneal inflammation, pressure on the right ureter, and mild hydronephrosis. Blood and urine cultures were negative. Chest X-ray was unremarkable. Empiric antibiotics were initiated but were discontinued several days later when a herpetic rash developed around her lips. At that point, acyclovir and glucocorticoids were administered.

Twelve days into her admission, the patient developed shortness of breath. A follow-up CT scan showed improvement in the retroperitoneal inflammation and hydronephrosis yet signs of heart failure were evident. Echocardiography demonstrated severe LV failure with severe mitral regurgitation and pulmonary hypertension. Troponin C levels were elevated. With a probable diagnosis of myocarditis secondary to SLE, the patient was transferred to the ICU.

During her prolonged stay in the ICU, the patient was treated with high doses of methylprednisolone, followed by broad spectrum antibiotics for fever and sepsis. Blood cultures were positive for ESBL Klebsiella and MRSA. In addition, she required mechanical ventilation during 10 days for presumed acute respiratory distress syndrome, as well as hemodialysis due to severe renal failure with volume overload. Eventually, her fever subsided and she was extubated, after which she was transferred back to the internal medicine department.

On her arrival in the department, she was dyspneic with O_2_ saturation of 85% on room air, with generalized edema and proximal muscle weakness. A ventilation/perfusion lung scan demonstrated low probability of pulmonary embolism. Repeat echocardiography showed severe LV failure with severe pulmonary hypertension. Of note, in addition to her declining physical condition, the patient was inclined to psychotic outbursts and unruly behavior, which were attributed at the time to SLE psychosis.

Due to her severe heart failure and psychotic episodes, both attributed to SLE, a decision was made to start treatment with high dose IVIg. The patient was treated with 0.4 g/kg IVIg per day for 5 consecutive days with dramatic results. One week later, she was able to start physical therapy. The edema completely disappeared and her psychotic episodes subsided. Follow-up echocardiography showed improvement in pulmonary hypertension, although evidence of heart failure remained.

### 2.5 Patient 5

This 35-year-old woman had been diagnosed a month previously with SLE by presenting with 4 of 11 ACR criteria,^[29]^ including: anemia, nephropathy, oral ulcers, and arthritis. She was being treated with prednisone and was admitted with a chief complaint of shortness of breath that started several hours prior to her admission. She also noted fever at home but denied chest pain. Her vital signs on admission were: temperature 38°C, blood pressure 156/111, heart rate 120/min, respiratory rate 18/min, 0_2_ saturation 93% on room air, 97% with 2 L nasal cannula. Hemoglobin was 8.0 g/dL, WBC count was 5.17 × 10^9^/L, and platelet count was 88 × 10^9^/L. A chest X-ray in the ED demonstrated centrally located bilateral alveolar infiltrates and bilateral pleural effusions. With a working diagnosis of pulmonary edema, secondary to a lupus flare, treatment was begun with IV corticosteroids, as well as broad spectrum IV antibiotics and furosemide. A non-contrast CT scan performed 2 days after admission showed a moderate pericardial effusion, moderate bilateral pleural effusions with surrounding atelectasis, multiple paratracheal nodules and bilateral perihilar infiltrates described as ground glass. Echocardiography revealed LV dysfunction with an EF of 35%, right ventricular hypokinesis, biatrial enlargement, moderate mitral regurgitation, mild to moderate tricuspid regurgitation, moderate pulmonary hypertension and a small pericardial effusion. It should be noted that a previous echocardiography, performed approximately 1 month earlier, demonstrated normal LV function. The patient continued to require large doses of furosemide, carvedilol, and IV nitroglycerin without significant abatement of symptoms. Her oxygen saturation on room air remained in the high 80s-low 90s. On the fifth day of admission, a decision was made to start treatment with high dose IVIg and she received 0.5 g/kg IVIg per day for 4 days. Thereafter, her condition improved dramatically and she no longer required oxygen by nasal cannula. Repeat echocardiography demonstrated marked improvement in LV function.

## 3 Discussion

IVIg is effective for treating several autoimmune conditions including some manifestations of SLE. However, it is usually reserved for cases when conventional therapies have failed or are contraindicated.

Evidence is accumulating that autoimmune processes are involved in the pathogenesis of cardiovascular diseases, specifically congestive heart failure (CHF) and cardiomyopathy.^[30,31]^ Several studies have shown that IVIg downregulates inflammatory responses in CHF patients and has potential beneficial effects on LVEF.^[31–33]^

In a placebo controlled, double blind study, Gullestad et al^[33]^ demonstrated that the antiinflammatory effect of IVIg in chronic CHF was significantly correlated with improvement in LVEF, suggesting a potential for an immune-modulating therapy in addition to optimal, conventional cardiovascular treatment regimens in CHF patients. This effect was found independent of the cause of heart failure. Beneficial effects of IVIg have also been suggested in the acute setting—in acute myocarditis and cardiomyopathy.^[34–38]^ McNamara et al^[34]^ reported a series of patients with new-onset dilated cardiomyopathy treated with high-dose IVIg, resulting in marked improvement of ventricular function. Nevertheless, in another placebo-controlled study by the same group,^[39]^ IVIg had no effect on 62 patients with recent-onset cardiomyopathy. We believe that a certain percentage of spontaneous improvement in recent-onset cardiomyopathy, accompanied by differences in the dosage schedule between studies (for example, maintenance therapy was given only to patients with chronic CHF) are accountable for the discrepant results between these studies. A recent meta-analysis conducted by Huang et al,^[38]^ concluded that IVIg therapy resulted in lower in-hospital mortality and superior recovery of left ventricular function in patients with acute myocarditis. Others have described the successful use of IVIg in peripartum cardiomyopathy^[40]^ and in viral cardiomyopathy.^[41]^ The use of IVIg for CHF and cardiomyopathy remains controversial.

LM is a severe cardiac manifestation of SLE, treated with high-dose corticosteroids, with or without other immunosuppressive therapy, in addition to standard cardiac management.^[22]^ Because SLE-related myocarditis is rare, there are few prospective studies, and management is based on isolated cases or small-series reports. Our belief that IVIg could have a central role in the management of LM/cardiomyopathy was based on the positive effects of IVIg on the ventricular function in cardiomyopathy and heart failure, along with its benefits for SLE, in general.

The 5 patients described in this report suffered from severe, acute cardiomyopathy, which can probably be attributed to SLE. Diagnosis was based on clinical manifestations and on electrocardiographic, echocardiographic and biochemical characteristics. Two patients underwent coronary angiography demonstrating no evidence of obstructive coronary disease; thus, ruling out thrombotic events, or vasculitis as the etiology of cardiomyopathy. Recent studies suggested that cardiac magnetic resonance (CMR) can assist in detection of myocardial ischemia in SLE patients, potentially caused by microvascular coronary dysfunction, in the absence of obstructive coronary artery disease.^[42]^ Due to limited access to CMR, these 5 patients did not undergo this test. The gold standard for confirming the diagnosis remains endomyocardial biopsy.^[43]^ However, this procedure is not routinely used because of its low sensitivity and potential complications. Thus, it was not performed in our patients. In our case series, the response to high dose IVIg therapy was fast. A single course of 2 g/kg delivered over 2 to 5 days (as recommended for autoimmune diseases) led to prolonged improvement in cardiac status.

Previous reports have demonstrated the beneficial effects of IVIg on LM,^[28,44–50]^ but the number of patients was small and none focused on IVIg as the main treatment strategy.

The largest recent report on LM was a multicenter, retrospective study conducted by Thomas et al,^[26]^ in which 29 patients were diagnosed with LM. Similarly, to our cases, most had elevated troponin, abnormal electrocardiogram, and echocardiography revealed low (≤45%) LVEF. Eight of the 29 patients with LM were treated with IVIg in addition to high-dose corticosteroids. Seven received a single perfusion (2 g/kg delivered over 3–5 days). One patient underwent repeated perfusions (monthly for 6 months). Overall, LVEF recovery rates were high: 81% exhibited an LVEF ≥55% at the end of a median follow-up of 37 months. Nevertheless, the authors compared outcomes in patients who received cyclophosphamide vs those who did not. Therefore, conclusions as to the effectiveness of IVIg treatment could not be drawn.

In a retrospective case series, Zawadowski et al^[49]^ described the treatment and outcome of 24 patients with LM. Only 1 received IVIg for 5 days. The choice of immunosuppressive regimen, including dosage and length of treatment was based on the treating physician's preference. Similarly, in a retrospective case-control study, Zhang et al^[50]^ reported that 12 of the 25 patients diagnosed with LM were treated with IVIg, with results similar to those of other immunosuppressive therapies. In contrast, Du Toit et al in a retrospective study in South Africa found that IVIg given to 3 of 28 patients with LM was not effective.^[51]^

IVIg has also been suggested as an effective therapy for myocarditis secondary to other autoimmune diseases^[31]^ such as dermato-polymyositis,^[52]^ adult-onset Still disease^[53–55]^ and Kawasaki disease.^[56]^

IVIg exhibits diverse mechanisms of action in autoimmune diseases, which include enhanced suppressor activity, Fc receptor blockade, complement regulation, B and T cell regulation and other mechanisms – all contributing to immunomodulation.^[31]^ The specific mechanism of immunomodulation in SLE is not completely understood but it involves the effect of IVIg on T and B cell intracellular signaling, the interferon signaling pathway and the disrupted elimination of immune complexes and other cellular debris.^[11,57]^

In recent years, there has been a surge in the development of novel molecules that target Fc receptors and may offer an alternative to IVIg.^[58]^ The use of these agents for autoimmune diseases including SLE is being investigated in clinical trials.

Another future potential therapy is based on the fact that glycosylation patterns of IgG in IVIg preparations interfere with its antiinflammatory response.^[57]^ In vivo sialylation is a potent, novel approach to attenuate harmful autoantibody-mediated inflammation through glycoengineering endogenous antibodies and converting them to antiinflammatory mediators.^[59]^

The current study has inherent limitations and interpretation of the findings is limited by aspects of the study design and the generalizability of the results. As mentioned above, neither myocardial biopsy nor CMR were undertaken to support our patients’ diagnoses, therefore, other causes for myocardial dysfunction could not be completely ruled out. Furthermore, treatment protocols differed and were dependent on the treating physician's decision. The sample size was small, as well. Nevertheless, we believe this series is of great interest because it emphasizes the important role of IVIg in acute lupus cardiomyopathy. Randomized control trials are needed to elucidate the actual effect of IVIg vs other immunosuppressive therapies.

In conclusion, our 5 cases emphasize the complicated diagnosis and management of cardiac involvement in SLE, as well as the beneficial role of IVIg treatment. IVIg is increasingly being used for the treatment of various autoimmune diseases and it appears to be relatively well-tolerated and safe. Our belief is that its high cost justifies its use, especially in severe cases, such as the 5 discussed in this report. Even though spontaneous recovery was a possibility for our patients, we believe that IVIg was a major contributing factor to the fast recovery of cardiac function.

## Author contributions

**Conceptualization:** Yehuda Shoenfeld, Yair Levy.

**Data curation:** Katya Meridor, Yair Levy.

**Supervision:** Yair Levy.

**Writing – original draft:** Katya Meridor, Yair Levy.

**Writing – review & editing:** Katya Meridor, Yehuda Shoenfeld, Oshrat Tayer-Shifman, Yair Levy.

## References

[1] Bayry J Negi VS Kaveri SV. Intravenous immunoglobulin therapy in rheumatic diseases. Nat Rev Rheumatol 2011;7:349–59. 21556030 10.1038/nrrheum.2011.61

[2] Galeotti C Kaveri SV Bayry J. IVIG-mediated effector functions in autoimmune and inflammatory diseases. Int Immunol 2017;29:491–8. 28666326 10.1093/intimm/dxx039

[3] Gelfand EW. Intravenous immune globulin in autoimmune and inflammatory diseases. N Engl J Med 2012;367:2015–25. 23171098 10.1056/NEJMra1009433

[4] Schroeder JO Zeuner RA Euler HH. High dose intravenous immunoglobulins in systemic lupus erythematosus: clinical and serological results of a pilot study. J Rheumatol 1996;23:71–5. http://www.ncbi.nlm.nih.gov/pubmed/8838511 http://www.ncbi.nlm.nih.gov/pubmed/8838511. Accessed November 7, 2019. 8838511

[5] Francioni C Galeazzi M Fioravanti A. Long-term i.v. Ig treatment in systemic lupus erythematosus. Clin Exp Rheumatol 1994; 2019;12:163–8. http://www.ncbi.nlm.nih.gov/pubmed/8039283 http://www.ncbi.nlm.nih.gov/pubmed/8039283. Accessed November 7,. 8039283

[6] Puddu P De Pità O Ruffelli M. Intravenous immunoglobulin therapy: modification of the immunofluorescence pattern in the skin of six patients with systemic lupus erythematosus. Arthritis Rheum 1996;39:704–5. 8630125 10.1002/art.1780390426

[7] Levy Y Sherer Y Ahmed A. A study of 20 SLE patients with intravenous immunoglobulin--clinical and serologic response. Lupus 1999;8:705–12. 10602441 10.1191/096120399678841007

[8] Sherer Y Kuechler S Scali JJ. Low dose intravenous immunoglobulin in systemic lupus erythematosus: analysis of 62 cases. Isr Med Assoc J 2008;10:55–7. 18300575

[9] Nieto-Aristizábal I Martínez T Urbano M-A. Treatment with intravenous immunoglobulins in systemic lupus erythematosus: a single-center experience with 63 patients. Lupus 2019;28:1566–70. 31653191 10.1177/0961203319883680

[10] Camara I Sciascia S Simoes J. Treatment with intravenous immunoglobulins in systemic lupus erythematosus: a series of 52 patients from a single centre. Clin Exp Rheumatol 2014;32:41–7. 24029366

[11] Zandman-Goddard G Levy Y Shoenfeld Y. Intravenous immunoglobulin therapy and systemic lupus erythematosus. Clin Rev Allergy Immunol 2005;29:219–28. 16391397 10.1385/CRIAI:29:3:219

[12] Mulhearn B Bruce IN. Indications for IVIG in rheumatic diseases. Rheumatol (United Kingdom) 2015;54:383–91. 10.1093/rheumatology/keu429 PMC4334686 25406359

[13] Zandman-Goddard G Krauthammer A Levy Y. Long-term therapy with intravenous immunoglobulin is beneficial in patients with autoimmune diseases. Clin Rev Allergy Immunol 2012;42:247–55. 21732045 10.1007/s12016-011-8278-7

[14] Hundt M Manger K Dörner T. Treatment of acute exacerbation of systemic lupus erythematosus with high-dose intravenous immunoglobulin [10]. Rheumatology 2000;39:1301–2. 11085821 10.1093/rheumatology/39.11.1301

[15] Aharon A Levy Y Bar-Dayan Y. Successful treatment of early secondary myelofibrosis in SLE with IVIG. Lupus 1997;6:408–11. 9175029 10.1177/096120339700600412

[16] Levy Y Sherer Y George J. Intravenous immunoglobulin treatment of lupus nephritis. Semin Arthritis Rheum 2000;29:321–7. 10805356 10.1016/s0049-0172(00)80018-9

[17] Boletis JN Ioannidis JPA Boki KA. Intravenous immunoglobulin compared with cyclophosphamide for proliferative lupus nephritis. Lancet 1999;354:569–70. 10470708 10.1016/S0140-6736(99)01575-5

[18] Milstone AM Meyers K Elia J. Treatment of acute neuropsychiatric lupus with intravenous immunoglobulin (IVIG): a case report and review of the literature. Clin Rheumatol 2005;24:394–7. 15662488 10.1007/s10067-004-1046-9

[19] Lim KS Cheong KL Tan CT. Periodic lateralized epileptiform discharges in neuropsychiatric lupus: association with cerebritis in magnetic resonance imaging and resolution after intravenous immunoglobulin. Lupus 2010;19:748–52. 20133346 10.1177/0961203309351539

[20] Tomer Y Shoenfeld Y. Successful treatment of psychosis secondary to SLE with high dose intravenous immunoglobulin. Clin Exp Rheumatol 1992;10:391–3. http://www.ncbi.nlm.nih.gov/pubmed/1395223 http://www.ncbi.nlm.nih.gov/pubmed/1395223. Accessed November 14, 2019. 1395223

[21] Perricone R De Carolis C Kröegler B. Intravenous immunoglobulin therapy in pregnant patients affected with systemic lupus erythematosus and recurrent spontaneous abortion. Rheumatology 2008;47:646–51. 18346976 10.1093/rheumatology/ken046

[22] Tanwani J Tselios K Gladman DD. Lupus myocarditis: a single center experience and a comparative analysis of observational cohort studies. Lupus 2018;27:1296–302. 29642752 10.1177/0961203318770018

[23] Doherty NE Siegel RJ. Cardiovascular manifestations of systemic lupus erythematosus. Am Heart J 1985;110:1257–65. 3907317 10.1016/0002-8703(85)90023-7

[24] Apte M McGwin G Vila LM. Associated factors and impact of myocarditis in patients with SLE from LUMINA, a multiethnic US cohort. Rheumatology 2007;47:362–7. 10.1093/rheumatology/kem371 18250089

[25] Miner JJ Kim AHJ. Cardiac manifestations of systemic lupus erythematosus. Rheum Dis Clin North Am 2014;40:51–60. 24268009 10.1016/j.rdc.2013.10.003

[26] Thomas G Aubart FC Chiche L. Lupus myocarditis: Initial presentation and longterm outcomes in a multicentric series of 29 patients. J Rheumatol 2017;44:24–32. 28042125 10.3899/jrheum.160493

[27] Comarmond C Cacoub P. Myocarditis in auto-immune or auto-inflammatory diseases. Autoimmun Rev 2017;16:811–6. 28572050 10.1016/j.autrev.2017.05.021

[28] Sherer Y Levy Y Shoenfeld Y. Marked improvement of severe cardiac dysfunction after one course of intravenous immunoglobulin in a patient with systemic lupus erythematosus. Clin Rheumatol 1999;18:238–40. 11206350 10.1007/s100670050091

[29] Tan EM Cohen AS Fries JF. The 1982 revised criteria for the classification of systemic lupus erythematosus. Arthritis Rheum 1982;25:1271–7. 7138600 10.1002/art.1780251101

[30] Becker NP Müller J Göttel P. Cardiomyopathy — An approach to the autoimmune background. Autoimmun Rev 2017;16:269–86. 28163240 10.1016/j.autrev.2017.01.012

[31] Nussinovitch U Shoenfeld Y. Intravenous immunoglobulin - Indications and mechanisms in cardiovascular diseases. Autoimmun Rev 2008;7:445–52. 18558360 10.1016/j.autrev.2008.04.001

[32] Aukrust P Yndestad A Damås JK. Inflammation and chronic heart failure-potential therapeutic role of intravenous immunoglobulin. Autoimmun Rev 2004;3:221–7. 15110235 10.1016/S1568-9972(03)00103-4

[33] Gullestad L Aass H Fjeld JG. Immunomodulating therapy with intravenous immunoglobulin in patients with chronic heart failure. Circulation 2001;103:220–5. 11208680 10.1161/01.cir.103.2.220

[34] McNamara DM Rosenblum WD Janosko KM. Intravenous immune globulin in the therapy of myocarditis and acute cardiomyopathy. Circulation 1997;95:2476–8. 9184576 10.1161/01.cir.95.11.2476

[35] Kishimoto C Shioji K Kinoshita M. Treatment of acute inflammatory cardiomyopathy with intravenous immunoglobulin ameliorates left ventricular function associated with suppression of inflammatory cytokines and decreased oxidative stress. Int J Cardiol 2003;91:173–8. 14559127 10.1016/s0167-5273(03)00002-0

[36] Tedeschi A Airaghi L Giannini S. High-dose intravenous immunoglobulin in the treatment of acute myocarditis. A case report and review of the literature. J Intern Med 2002;251:169–73. 11905592 10.1046/j.1365-2796.2002.00929.x

[37] Kishimoto C Shioji K Hashimoto T. Therapy with immunoglobulin in patients with acute myocarditis and cardiomyopathy: Analysis of leukocyte balance. Heart Vessels 2014;29:336–42. 23702697 10.1007/s00380-013-0368-4

[38] Huang X Sun Y Su G. Intravenous immunoglobulin therapy for acute myocarditis in children and adults a meta-analysis. Int Heart J 2019;60:359–65. 30745539 10.1536/ihj.18-299

[39] McNamara DM Holubkov R Starling RC. Controlled trial of intravenous immune globulin in recent-onset dilated cardiomyopathy. Circulation 2001;103:2254–9. 11342473 10.1161/01.cir.103.18.2254

[40] Bozkurt B Villaneuva FS Holubkov R. Intravenous immune globulin in the therapy of peripartum cardiomyopathy. J Am Coll Cardiol 1999;34:177–80. 10400008 10.1016/s0735-1097(99)00161-8

[41] Maisch B Alter P. Treatment options in myocarditis and inflammatory cardiomyopathy: focus on i.v. immunoglobulins. Herz 2018;43:423–30. 29947834 10.1007/s00059-018-4719-x PMC6096625

[42] Ishimori ML Martin R Berman DS. Myocardial ischemia in the absence of obstructive coronary artery disease in systemic lupus erythematosus. JACC Cardiovasc Imaging 2011;4:27–33. 21232700 10.1016/j.jcmg.2010.09.019

[43] Caforio ALP Pankuweit S Arbustini E. Current state of knowledge on aetiology, diagnosis, management, and therapy of myocarditis: a position statement of the European Society of Cardiology Working Group on Myocardial and Pericardial Diseases. Eur Heart J 2013;34:2636–48. 2648a-2648d. 23824828 10.1093/eurheartj/eht210

[44] Disla E Rhim HR Reddy A. Reversible cardiogenic shock in a patient with lupus myocarditis. J Rheumatol 1993;20:2174 http://www.ncbi.nlm.nih.gov/pubmed/8014958 http://www.ncbi.nlm.nih.gov/pubmed/8014958. Accessed December 14 2019. 8014958

[45] Charhon N Bernard C Richard JC. Off-label use of intravenous immunoglobulin therapy in the treatment of lupus myocarditis: two case reports and literature review. Rev Med Interne 2017;38:204–9. 27263120 10.1016/j.revmed.2016.05.005

[46] Micheloud D Calderon M Caparrros M. Intravenous immunoglobulin therapy in severe lupus myocarditis: Good outcome in three patients. Ann Rheum Dis 2007;66:986–7. 17576788 10.1136/ard.2006.058784 PMC1955109

[47] Barnado A Kamen DL. Myocarditis successfully treated with intravenous immunoglobulin in a patient with systemic lupus erythematous and myositis. Am J Med Sci 2014;347:256–7. 24521767 10.1097/MAJ.0000000000000232

[48] Suri V Varma S Joshi K. Lupus myocarditis: marked improvement in cardiac function after intravenous immunoglobulin therapy. Rheumatol Int 2010;30:1503–5. 19697032 10.1007/s00296-009-1098-x

[49] Zawadowski GM Klarich KW Moder KG. A contemporary case series of lupus myocarditis. Lupus 2012;21:1378–84. 22892209 10.1177/0961203312456752

[50] Zhang L Zhu YL Li MT. Lupus myocarditis: a case–control study from China. Chin Med J (Engl) 2015;128:2588–94. 26415795 10.4103/0366-6999.166029 PMC4736867

[51] Du Toit R Herbst PG van Rensburg A. Clinical features and outcome of lupus myocarditis in the Western Cape, South Africa. Lupus 2017;26:38–47. 27225211 10.1177/0961203316651741

[52] Cuny C Eicher JC Collet E. Dilated cardiomyopathy disclosing dermatopolymyositis. Management. Ann Cardiol Angeiol (Paris) 1993;42:155–8. 8498803

[53] Gerfaud-Valentin M Sève P Iwaz J. Myocarditis in adult-onset still disease. Med (United States) 2014;93:280–9. 10.1097/MD.0000000000000112 PMC4602418 25398063

[54] Neto NSR Waldrich L de Carvalho JF. Adult-onset Still's disease with pulmonary and cardiac involvement and response to intravenous immunoglobulin. Acta Reum Port 2009;34:628–32. 20087268

[55] Kuek A Weerakoon A Ahmed K. Adult-onset Still's disease and myocarditis: Successful treatment with intravenous immunoglobulin and maintenance of remission with etanercept. Rheumatology 2007;46:1043–4. 17449487 10.1093/rheumatology/kem066

[56] Dionne A Dahdah N. Myocarditis and Kawasaki disease. Int J Rheum Dis 2018;21:45–9. 29105303 10.1111/1756-185X.13219

[57] Martínez T Garcia-Robledo JE Plata I. Mechanisms of action and historical facts on the use of intravenous immunoglobulins in systemic lupus erythematosus. Autoimmun Rev 2019;18:279–86. 30639648 10.1016/j.autrev.2018.10.002

[58] Zuercher AW Spirig R Baz Morelli A. Next-generation Fc receptor–targeting biologics for autoimmune diseases. Autoimmun Rev 2019;18:102366. 31404703 10.1016/j.autrev.2019.102366

[59] Pagan JD Kitaoka M Anthony RM. Engineered sialylation of pathogenic antibodies in vivo attenuates autoimmune disease. Cell 2018;172:564–77. e13. 29275858 10.1016/j.cell.2017.11.041 PMC5849077

